# HPV infection screening using surface plasmon resonance in women from Kunming (Southwest China)

**DOI:** 10.17305/bjbms.2019.4352

**Published:** 2020-02

**Authors:** Yang Liu, Yue Quan, Changjun Xu, Yajuan Huang, Daizhu Li, Qing Qing, Chunyi Sun, Honglin Zhou

**Affiliations:** 1 Department of Reproductive, The Second Affiliated Hospital of Kunming Medical University, Kunming Yunnan, China; 2 Department of Obstetrics and Gynecology, The First Affiliated Hospital of Xiamen University, Xiamen Fujian, China

**Keywords:** Cervical cancer, screening, human papilloma virus, HPV, surface plasmon resonance, SPR, squamous intraepithelial lesions, carcinoma *in situ*, cervical intraepithelial neoplasia, CIN

## Abstract

No study examined the frequency of human papillomavirus (HPV) genotypes by surface plasmon resonance (SPR) in Southwest China. This was a cross-sectional survey (The Second Affiliated Hospital of Kunming Medical University, 10/2010 to 12/2011) in 150 patients who were hospitalized or volunteered for cervical cancer (CC) screening. A HPV typing kit was used to detect 24 types of HPV by the SPR technique. The HPV-positive rate was 34.8% in women with normal cytology and 92.9% in women with CC. The frequency of HPV16 increased from 9.4% for women with normal cytology to 28.9% for cervical intraepithelial neoplasia (CIN)1, 41.4% for CIN2, 54.1% for CIN3, and 71.4% for CC (*p* < 0.001). The frequency of HPV18 increased from 0% for women with normal cytology to 2.6% for CIN1, 3.4% for CIN2, 5.4% for CIN3, and 21.4% for CC (*p* = 0.03). HPV40 was only found in one patient with CC (*p* = 0.04). There was no relation between HPV genotype and women’s age. In Kunming (Southwest China), the frequency of HPV infection was 74.0% among women who underwent CC screening. HPV16 and HPV18 were the two most frequent genotypes. SPR could be of value for the screening of HPV infection.

## INTRODUCTION

The human papillomavirus (HPV) is the causative agent for cervical cancer (CC) [1]. Nearly all sexually active women are infected with HPV in their lifetime, but only 10% of them will develop persistent infection [2]. Over 200 different HPV genotypes are known and about 40 of them are more susceptible to genital tract transmission and infection [3,4]. Among those 40 genotypes, 14 (HPV 16, 18, 31, 33, 35, 39, 45, 51, 52, 56, 58, 59, 66, and 68) are deemed at high risk of CC [5,6]. Persistent infections with high-risk HPVs contribute to the incidence of CC [2,7,8]. In 2012, about 4.5% of all new cancer cases worldwide were attributed to HPV, with 80% of those cases being CC [9-11]. China represents 62,000 of the new CC cases (11.7% of the worldwide cases) and 30,000 deaths from CC (11.4%) [3,9,11].

The worldwide prevalence of HPV infection is estimated at 11.7%, but it varies widely among geographical regions and age groups [3,12,13]. Regarding age, the highest prevalence, around 24.0%, is found in women <25 years old [12,13]. Geographically, the highest prevalence rates are found in Oceania and Africa [3,12,13]. Regarding the genotypes, the worldwide prevalence of HPV16 is 3.2%, followed by HPV18 (1.4%), HPV52 (0.9%), HPV31 (0.8%), and HPV58 (0.7%) [3,12,13]. In China, HPV positivity rates vary widely among regions, from 9.9% to 27.5%; the prevalence of HPV 16 and 18 is a little lower than in the world, and the most prevalent genotypes are HPV 16, 18, 52, 58, and 33 [14-16].

CC screening aims to decrease the morbidity and mortality of the disease in women. A systematic review showed that CC screening appears to reduce the incidence of invasive CC [17]. It has been estimated that cervical cytology screening in the United States (US) has reduced the incidence and mortality of CC by 50% over the past 30 years [18]. Cervical cytology combined with HPV DNA testing appears to have high sensitivity but low specificity for detecting cervical intraepithelial neoplasia (CIN)3 or worse [19] and for identifying women with low-grade lesions requiring a biopsy [20]. This cotesting has been recommended by the American Cancer Society since 2012 [21]. The European guidelines have recognized the inclusion of high-risk HPV in primary screening programs since 2015 [22].

Surface plasmon resonance (SPR) is a novel technology for a direct and label-free analysis of organic compounds and DNA. SPR can detect 24 HPV genotypes in one reaction [23,24].

No study examined the epidemiology of HPV by SPR in Southwest China up until now. Therefore, the present study aimed to identify HPV infection of female patients in Kunming by the SPR technique and explore the correlation with CC.

## MATERIALS AND METHODS

### Study design and patients

This was a cross-sectional survey that was carried out at the Gynecology Outpatient Clinic and Gynecology Inpatient Department of the Second Affiliated Hospital of Kunming Medical University in 150 patients who were hospitalized or volunteered for CC screening, between October 2010 and December 2011. The study was approved by the Ethics Committee of the Second Affiliated Hospital of Kunming Medical University. All women provided written informed consent.

The inclusion criteria were: 1) adult women; 2) women who had sex without pregnancy; 3) no previous treatment for cervical and vaginal diseases; 4) no vaginal operation or sexual intercourse within 72 h before examination; and 5) informed consent was obtained.

### Sample collection

The cell brush was inserted into the external os of the cervix, at the junction of the squamous and columnar epithelium; it was rotated evenly 3–5 times using the external os of the cervix as the center. The cervical brush was taken out, and the brush head was put into the cervical exfoliated cell preservation solution and evenly mixed to dissolve the specimen into the preservation solution (Liquid-based Cell Processing, Smearing and Staining Kit, Taipu Bioscience Co., Ltd., Xiamen, China). All subjects were tested for cervical lesions using cervical sedimentation type ThinPrep liquid-based cytology (LBC). The remaining cells in the preservation solution were extracted by the SPR technique for the detection of HPV. Cervical biopsy was performed under colposcopy for subjects whose LBC ≥atypical squamous cells of undetermined significance (ASCUS) or for those with high-risk HPV detected by the SPR method.

### SPR detection

A HPV typing kit (Beijing GP Medical Technology Co., Ltd., Beijing, China) was used for detecting 24 HPV types, including 16 high-risk types (16, 18, 52, 58, 31, 33, 56, 59, 66, 45, 53, 39, 51, 68, 35, and 81) and 8 low-risk types (6, 11, 42, 70, 44, 43, 54, and 40), according to the manufacturer’s instructions [25]. The kit includes a chip containing probes for the 24 types of HPV, a control probe for the β-actin gene, and a negative control probe. Extracted DNA from the specimens is amplified by PCR and denatured together with β-actin DNA (amplified in parallel) at 95°C for 3 min before being injected into the channel of the chip. After hybridization at 40°C for 15 min, PCR mixtures are analyzed using the W2600 system (GP Medical Technologies Co., Ltd., Beijing, China). In a successful reaction, the positive control probe spot yields a positive hybridization resonance unit (RU), the negative control probe spot a negative hybridization RU, and the rest of HPV probe spots different hybridization RUs, depending on the HPV status in each specimen.

### LBC detection

The Liquid-based Cell Processing, Smearing and Staining Kit from Taipu Bioscience Co., Ltd. (Xiamen, China) was used according to the manufacturer’s instructions. Preservation buffer was vortexed for 30 s to resuspend cells. Gradient centrifugation buffer (4 ml) was added to a centrifuge tube together with 8 ml of cell preservation buffer. The mixture was centrifuged at 1080 rpm for 2 min. The top 8 ml were discarded and the tube was centrifuged again at 2000 rpm for 10 min. After the supernatant was discarded, buffer solution (500–800 µl) was added to the bottom and vortexed for 15 ± 5 s. Then, 30 µl of this suspension was transferred to a slide, and the cells were left to adhere for 10 min. The slide was rinsed, stained twice with 500 µl of hematoxylin for 90–120 s each time, and rinsed with rinsing solution after each staining. The slide was then stained with 500 µl of Papanicolaou staining solution for 90–120 s. The slides were mounted with optical resin and observed under a microscope. The results were classified as: 1) no intraepithelial lesion or malignant lesion (NILM), which meant that no abnormal squamous epithelial cells or abnormal glandular epithelial cells were found; NILM also included non-neoplastic findings such as inflammatory or reactive changes caused by various microbial infections; 2) low-grade squamous intraepithelial lesions (LSILs), which referred to mild squamous epithelial cell morphological abnormality; the majority of the patients were diagnosed with squamous epithelial dysplasia or CIN1 and there might also be cervical CIN2, CIN3, or some reactive changes; and 3) high-grade squamous intraepithelial lesions (HSILs), which referred to moderate to severe dysplasia and carcinoma *in situ* (CIS), CIN2, and CIN3, showing abnormal cells with an increased ratio of nucleus to cytoplasm. “Positive” LBC included all results other than NILM.

### Pathological examination

The high-risk group found by screening was examined by colposcopy and cervical biopsy. The specimens were fixed with 10% formalin, routinely embedded in paraffin, sectioned, stained with hematoxylin and eosin (H&E), and observed by light microscopy.

### Diagnostic criteria for pathological examination

LSIL (CIN1) presented as nuclear pleomorphism and deep staining involving the lower one-third of the epithelium. Nuclear chromatin was irregular, and the nucleoli were not conspicuous. The mitotic activity was increased in the lower one-third of the epithelium. Most cells displayed the HPV effect. Flat condyloma acuminatum was considered to be LSIL.

HSIL (CIN2 or CIN3) and CIS presented as nuclear pleomorphism and deep staining involving the lower two-third of the epithelium (HSIL; CIN2) or the full layer (HSIL; CIN3; CIS). Nuclear chromatin was irregular and the nucleoli were not conspicuous. There was a high nuclear-cytoplasm ratio with the presence of atypical mitosis. Binuclear and multinuclear cells were common but less than in LSIL. HPV was occasionally present in lesions and HPV was more common near lesions.

### Data collection

Demographic characteristics (age, marital status, educational level, smoking, and alcohol), reproductive health characteristics (menopause, number of sexual partners, and extramarital affair), birth history (parity), and clinical characteristics (history of sexually transmitted disease, family history of cancer, history of cervical screening, *Chlamydia trachomatis* status, and HPV status) of patients were collected.

### Statistical analysis

Continuous data were presented as mean ± standard deviation. The categorical data were presented as frequencies and analyzed using the Chi-square test or Fisher’s exact test where appropriate. All statistical analyses were performed using SPSS for Windows, Version 16.0. (SPSS Inc., Chicago, IL, US). A value of *p* < 0.05 was considered statistically significant.

## RESULTS

### Characteristics of the patients

Table 1 presents the sociodemographic, clinical, and anamnestic characteristics of the examined population according to the diagnosis of cervical pathology. Most women were from rural areas, <39 years of age, married, married before <25 years of age, with higher education, non-smokers, non-drinkers, and not in menopause. None reported having extramarital affairs. Most women did not have a history of sexually transmitted disease, familial history of cancer, nor did they undergo cervical screening. The HPV-positive rate increased from 34.8% for women with normal cytology to 92.9% for women with CC diagnosis (Table 1).

**TABLE 1:**
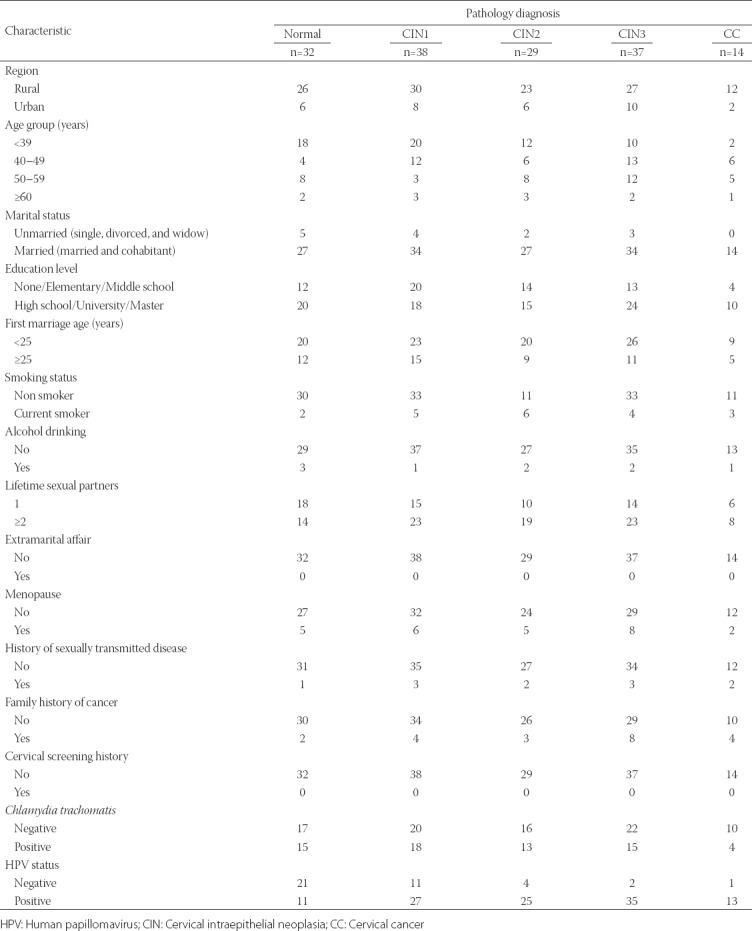
Sociodemographic, clinical, and anamnestic characteristics of the examined population according to the diagnosis of cervical pathology

### HPV genotype and cervical lesions

The frequency of HPV16 increased from 9.4% for women with normal cytology to 28.9% for CIN1, 41.4% for CIN2, 54.1% for CIN3, and 71.4% for CC [*p* < 0.001] (Table 2). The frequency of HPV18 increased from 0% for women with normal cytology to 2.6% for CIN1, 3.4% for CIN2, 5.4% for CIN3, and 21.4% for CC (*p* = 0.03). HPV40 was only found in one patient with CC (*p* = 0.04). There were no differences among lesion types regarding the remaining 21 HPV genotypes (all *p* > 0.05).

**TABLE 2:**
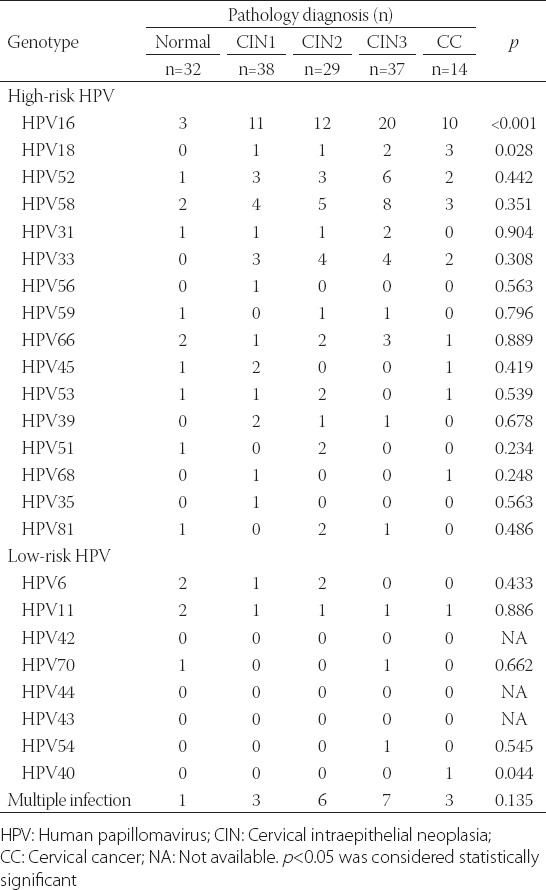
Distribution of HPV types according to the severity of cervical lesions

### HPV genotype and women’s age

Table 3 shows that there was no relation between HPV genotype and women’s age (*p* = 0.40).

**TABLE 3:**
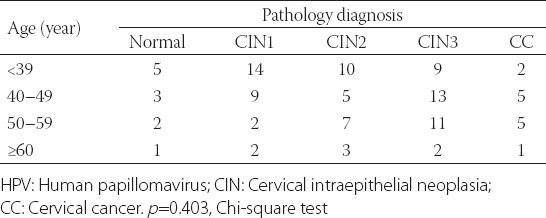
HPV infection rate according to the age and severity of cervical lesions

## DISCUSSION

Up until now, no study has examined the frequency of HPV genotypes by SPR in Southwest China. Therefore, our objective was to identify HPV infection of female patients in Kunming by the SPR technique and explore the correlation with CC. The results indicated that HPV16 and HPV18 were the two most frequent genotypes. SPR may be useful for the detection of HPV infection in women undergoing CC screening.

In the present study, the frequency of HPV infection was 74% among 150 women who underwent CC screening in Kunming. This rate is considerably higher compared to previous studies from China [14-16] and could be biased by a number of factors. Half of the included patients were hospitalized because of vaginal symptoms, which could increase the likelihood of finding cervical lesions and detecting HPV, as shown by the high number of patients with CC (14/150). Most women were from rural areas, and there is a possibility that those women visited the hospital when the symptoms were getting worse. In addition, cervical lesions are related to the age of women [13,26] and the age at the first sexual intercourse [27]. Occurrence of cervical lesions is also associated with smoking, the number of sexual partners, family history of cancer, and menopause [28,29]. Therefore, the rate in this study cannot be used as a measure of the frequency of HPV infection in Kunming, and additional studies are necessary to determine the exact epidemiology of HPV in Southwest China.

In the present study, HPV16 and HPV18 were the two most common genotypes, with increasing frequencies from normal cervix to CC. A cross-sectional 5-year study of non-vaccinated women from Chongqing (Southwest China) showed that HPV16 and HPV18 were indeed the most frequent genotypes and that their frequency increased with lesion severity [14]. A review of epidemiological studies from China showed that HPV16 was the most frequent genotype [15]. Other studies from China also reported the predominance of HPV16 and HPV18 [13,30]. HPV16 is the most frequent HPV genotype associated with cervical lesions in the world [31]. The distribution of HPV genotypes, particularly HPV16, is directly related to the severity of cervical lesions [32], as observed in the present study. Among women with CIN2 and CIN3, the most frequent genotypes were still HPV16 and HPV18. An international study suggested that HPV-based CC screening should focus on HPV16 and HPV18 [33], supporting our results.

A significant proportion of women were HPV-positive but had negative cytology. This probably reflects low viral loads in the setting of early infection. As SPR is a sensitive method for the detection of HPV [23,24], these women were determined as being HPV-positive. In addition to good sensitivity and reliability, SPR has several other advantages. It does not require any labeling and the measurement is direct. The chips contain probes for 24 HPV genotypes, and the signal is detected when a probe hybridizes to DNA of a specific sequence. The sample can be detected directly, without complex preparation [23,24]. Therefore, SPR could be used for the automated high-throughput analysis of HPV genotypes in CC screening. SPR has been shown to be as reliable as other tests for HPV detection [34].

The present study has limitations. It was a single-center study of a small number of women seeking HPV screening. In addition, the SPR results were not confirmed by sequencing, because it is a time-consuming and expensive method.

## CONCLUSION

In Kunming (Southwest China), the frequency of HPV infection was 74.0% among women who underwent CC screening, and HPV16 and HPV18 were the two most frequent genotypes. SPR could be useful for detection of HPV infection in women undergoing CC screening.
